# Is Remnant Preservation in Anterior Cruciate Ligament Reconstruction Superior to the Standard Technique? A Systematic Review and Meta-Analysis

**DOI:** 10.1155/2019/1652901

**Published:** 2019-12-11

**Authors:** Han Wang, Ziming Liu, Yuwan Li, Yihang Peng, Wei Xu, Ning Hu, Wei Huang

**Affiliations:** ^1^Department of Joint Surgery, First Affiliated Hospital of Chongqing Medical University, Chongqing, China; ^2^Institute of Sports Medicine Beijing Key Laboratory of Sports Injuries, Peking University Third Hospital, Beijing, China; ^3^Department of Joint Surgery, First Affiliated Hospital of Zunyi Medical University, Zunyi, China; ^4^First College of Clinical Medicine, Chongqing Medical University, Chongqing, China

## Abstract

**Purpose:**

This is a systematic review and meta-analysis of current evidence that aims at comparing the clinical outcomes of remnant-preserving anterior cruciate ligament reconstruction (ACLR) and standard ACLR.

**Methods:**

A systematic review of randomized controlled studies and cohort studies comparing remnant-preserving ACLR with standard ACLR with a minimum level of evidence of II was performed. Studies were included by strict inclusion and exclusion criteria. Extracted data were summarized as preoperative conditions, postoperative clinical outcomes, and postoperative complications. When feasible, meta-analysis was performed with RevMan5.3 software. Study methodological quality was evaluated with the modified Coleman methodology score (CMS).

**Results:**

Eleven studies (*n* = 466 remnant-preserving and *n* = 536 standard) met the inclusion criteria. The mean modified CMS for all included studies was 85.8 (range: 77–92 on a 100-point scale). In total, 466 patients underwent remnant-preserving ACLR by 3 different procedures: standard ACLR plus tibial remnant tensioning (*n* = 283), selective-bundle augmentation (*n* = 49), and standard ACLR plus tibial remnant sparing (*n* = 134). Remnant-preserving ACLR provided a superior outcome of postoperative knee anterior stability (WMD = −0.42, 95% CI, −0.66, −0.17; *P* < 0.01) and Lysholm score (WMD = 2.01, 95% CI, 0.53 to 3.50; *P* < 0.01). There was no significant difference between the two groups with respect to second-look arthroscopy (OR = 1.38, 95% CI, 0.53, 3.62; *P*=0.51), complications (OR = 1.24 95% CI, 0.76, 2.02; *P*=0.39), International Knee Documentation Committee (IKDC) subject scores, IKDC grades, Lachman test, and pivot-shift test.

**Summary/conclusion:**

Remnant-preserving ACLR promotes similar graft synovial coverage and revascularization to standard ACLR. Equivalent or superior postoperative knee stability and clinical scores were observed for remnant-preserving ACLR compared with standard ACLR. No significant difference in the total complication rate between the groups was evident.

## 1. Introduction

Anterior cruciate ligament (ACL) reconstruction (ACLR) has become a popular and effective surgery for the management of ACL injury [1–4]. However, the reinjury rate is still high, and a number of patients with poor clinical outcomes are observed at long-term follow-up [5, 6]. To achieve better knee stability and clinical outcomes, remnant-preserving ACLR, with its potential advantages of promoting faster graft revascularization and maturation, has been studied and compared to standard ACLR.

Many histological and animal studies have confirmed that ACL remnants retain a well-vascularized synovial sheet, numerous fibroblasts and myofibroblasts, and mechanoreceptors [7–13]. Some authors claim that remnants can accelerate the process of synovial coverage and revascularization and enhance the biomechanical properties of grafts in animals [8, 14]. However, many studies [15–25] have reported inconsistent clinical outcomes when comparing remnant-preserving ACLR to standard ACLR. Some studies [17, 22] have reported better arthroscopic evaluations and clinical outcomes for remnant-preserving ACLR. Other studies [16, 24] have found that remnant preservation can induce an increased incidence of postoperative extension loss. Several previous reviews have summarized these results. Papalia et al. [26] found significant postoperative improvements in patients undergoing remnant-preserving ACLR. Hu et al. [27] reported that the short-term clinical outcomes of patients with remnant-preserving ACLR are comparable to those of patients with standard ACLR. Two meta-analysis reviews [28, 29] reported similar clinical results between remnant-preserving ACLR and standard ACLR. However, previous systematic reviews are limited by their inclusion of a low level of evidence-based research. Based on previous studies, this review included new and high-quality studies with level I or level II evidence to perform a systematic review of the techniques and a meta-analysis of the functional and objective outcomes after remnant-preserving ACLR versus standard ACLR.

In clinical practice, the point of focus is not just the restoration of the biomechanics of the ACL by surgery; the biological healing of the ACL is a more important factor. At present, clinical studies show inconsistent results in graft healing by remnant-preserving ACLR [16, 17, 22, 24]. Therefore, the debate continues as to whether remnant-preserving ACLR promotes better graft healing than standard ACLR.

This is a systematic review and meta-analysis of current evidence that aims at comparing the clinical outcomes of remnant-preserving anterior cruciate ligament reconstruction (ACLR) and standard ACLR. We hypothesized that remnant-preserving ACLR could produce superior clinical outcomes to those of standard ACLR.

## 2. Methods

### 2.1. Literature Search

A systematic search of 4 databases, namely, PubMed, EMBASE, Medline Ovid, and Cochrane Library, was performed by two authors independently on December 10, 2018. The base terms used in each search included “anterior cruciate ligament remnant,” “ACL augmentation,” “ACL preservation,” “ACL stump,” “selective ACLR,” and “ACL remnant reconstruction.” The levels of evidence, namely, I and II (according to the Oxford Centre for Evidence-Based Medicine used by the Journal of Bone & Joint Surgery American Volume and Arthroscopy [30]), were reviewed for study inclusion. Two authors independently selected all articles by reviewing full-text reports according to the inclusion and exclusion criteria. Any disagreements between the two authors at the stage of inclusion were resolved through discussion with the corresponding author.

### 2.2. Study Selection

Studies were considered eligible if they met the following criteria: an adequate description of the remnant preservation technique for ACLR; reports of both preoperative conditions and postoperative clinical outcomes after primary remnant-preserving ACLR; level I or II evidence; studies that included subjective and objective outcomes; written in English; use of human subjects; and a study publication or in-press online date between January 1, 2000, and December 10, 2018. The exclusion criteria for this article are as follows: the follow-up period was less than one year; level III or IV evidence; study that does not directly compare the outcome of remnant-preserving ACLR and standard ACLR; and operative interventions were not described in the article. If the same population was included in more than one study, we included the study with the longest duration of follow-up.

### 2.3. Quality Assessment

The modified Coleman methodology score (CMS) [31], which comprises a 10-criterion validated score, was used to assess the methodological quality of each article by 2 authors. Each of the 10 criteria was scored to generate a total score between 0 and 100. A high score indicates a study design that largely avoids the influences of chance, different biases, and confounding factors.

### 2.4. Data Extraction

The extracted data were compared and discussed to meet consistency by all authors. Data extracted from each study included in this review were summarized as the (1) preoperative conditions, (2) postoperative clinical outcomes, and (3) postoperative complications of patients after remnant-preserving ACLR or after standard ACLR.

Each study was divided into 2 groups, namely, patients undergoing remnant-preserving ACLR and those undergoing standard ACLR. The postoperative outcomes and complications in the 2 groups of each study were proactively assessed and compared, which provided evidence to evaluate the effects of remnant-preserving ACLR. The items of the preoperative condition included (1) time from injury to surgery, (2) pattern of ACL rupture, (3) amount of ACL remnant, (4) type of ACL graft, and (5) surgical procedure (Table 1). The items of postoperative clinical outcome and complications included (1) time of follow-up, (2) stability and functional outcomes, (3) graft revascularization process, (4) proprioceptive testing, and (5) postoperative complications in all studies (Table 2).

### 2.5. Statistical Analysis

Continuous variable data (e.g., Lysholm scores) were collected as mean ± standard deviation from the mean. The differences were reported as weighted mean differences (WMDs). Dichotomous data (e.g., IKDC grade A or B vs. grade C or D) were reported as odds ratios. Two types of data were presented with 95% confidence intervals (CIs). When feasible, meta-analysis was performed with RevMan5.3 software (the Nordic Cochrane Centre, the Cochrane Collaboration, Copenhagen, Denmark). Random-effects models rather than the fixed-effects models were chosen to combine studies. Because random-effects models properly take into account heterogeneity when a few studies are combined, such as differences in study design. Significance was set at *P* < 0.05. Heterogeneity was assessed using *I*^2^. The values of *I*^2^ <25%, 50%, and >75% were interpreted as small, moderate, and high levels of heterogeneity, respectively. For quantitative syntheses including randomized controlled trials and prospective cohort studies, subgroup meta-analyses were presented for each study type group.

## 3. Results

### 3.1. Literature Search

**Figure 1:**
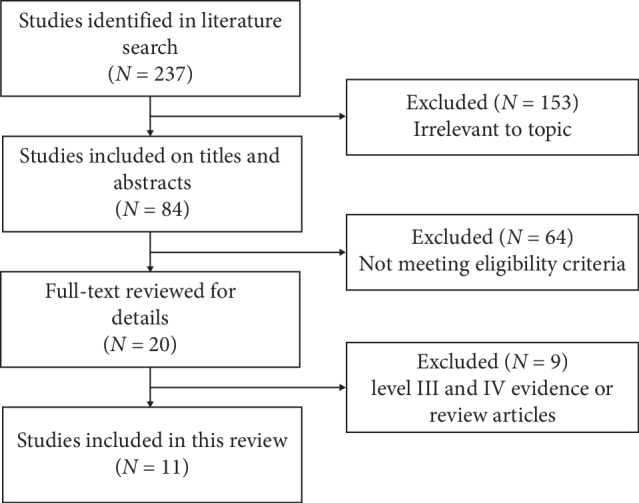
Flowchart of articles during the selection process.

A total of 237 relevant articles were initially identified according to the search strategy. One hundred fifty-three were excluded after reviewing the title because they were irrelevant to the topic. Sixty-four were excluded after reviewing the abstract. Nine were excluded according to low-level evidence or review articles. Finally, eleven high-level evidence proactive articles were included in this systematic review.  Figure 1 illustrates the search strategy for this review. The features of the levels of evidence for each included study are listed in Table 3.

### 3.2. Quality Assessment

All studies [15–25] that are included in this review are RCTs or prospective cohort studies, which provide strong assurance of study quality. The outcome criteria of the included study were clearly defined and reported good reliability, which included a subjective scoring system, physical examination, and second-look operation in partial patients. Each study had a minimal 1-year follow-up time with *a* > 80% recruitment rate. The mean modified CMS for all included studies was 85.8 (ranging from 77 to 92). The CMS scores and the detailed CMS of each study are shown in Tables 3 and 4, respectively.

### 3.3. Data Abstraction

In total, 466 patients underwent remnant-preserving ACLR by 3 different procedures: standard ACLR plus tibial remnant tensioning (*n* = 283), selective-bundle augmentation (*n* = 49), and standard ACLR plus tibial remnant sparing (*n* = 134). Surgical descriptions are presented in Table 5 [32–34].

The outcomes of patients after remnant-preserving ACLR (*n* = 466) and after standard ACLR (*n* = 536) were directly compared and included knee stability, clinical scoring system, and graft status.

### 3.4. Clinical Scoring System

**Figure 2:**
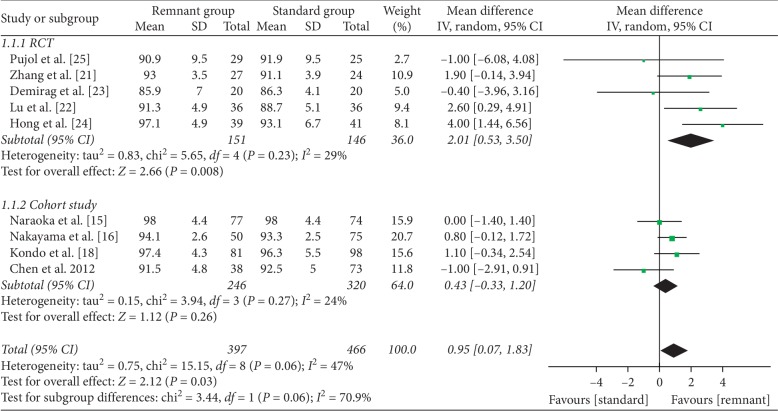
Forest plot for Lysholm scores. CI: confidence interval; IV: inverse variance.

Lysholm score was reported in nine studies (5 RCT and 4 cohort studies) [15, 16, 18, 21–25]. The pooled difference in mean postoperative value in RCT was 2.01 (95% CI, 0.53 to 3.50; *P* < 0.01) with moderate heterogeneity (*I*^2^ = 29%), in favor of the R group (remnant-preserving ACLR group). The pooled difference in the mean score in cohort studies was 0.43 (95% CI, −0.33 to 1.20; *P*=0.26), and no difference was found between the groups (Figure 2).

**Figure 3:**
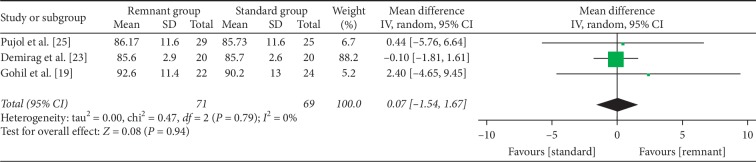
Forest plot for IKDC subject scores. CI: confidence interval; IV: inverse variance.

International Knee Documentation Committee (IKDC) subject scores were conducted in three RCT studies [19, 23, 25]. No significant difference was found between the two groups with respect to IKDC subject scores (WMD = 0.07, 95% CI, −1.54, 1.67; *P*=0.94) (Figure 3).

**Figure 4:**
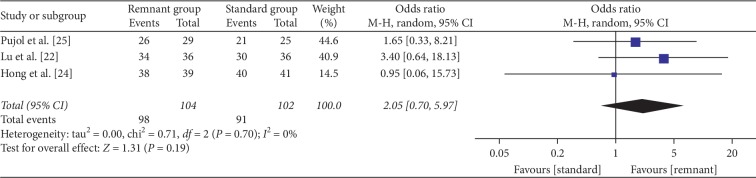
Forest plot for IKDC grades. CI: confidence interval; MH: Mantel–Haenszel.

Three studies were reviewed with respect to IKDC grades [22, 24, 25]. Superior results were defined as IKDC grade A or B. There was no significant difference between the two groups (OR = 2.05, 95% CI, 0.70, 5.97; *P*=0.19) (Figure 4).

### 3.5. Knee Stability

**Figure 5:**
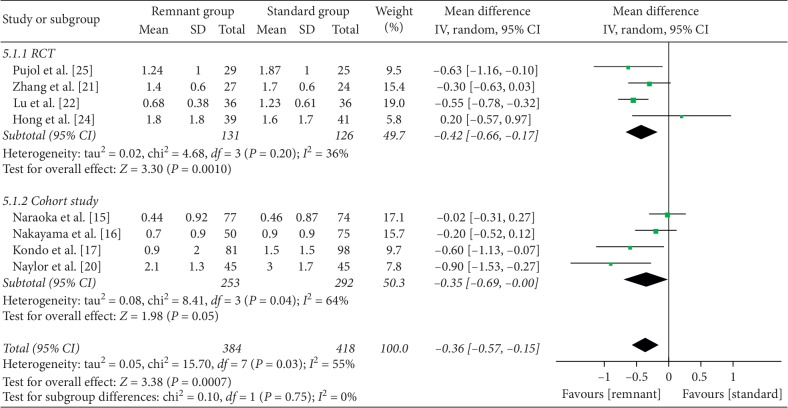
Forest plot for arthrometer measurements. CI: confidence interval; IV: inverse variance.

Anterior laxity was evaluated with a KT-1000/2000 arthrometer or the laxity Rolimeter in eight studies (4 RCT and 4 cohort studies) [15–17, 19–22, 24, 25]. A significant difference was found in arthrometer measurements in favor of the R group when evaluating RCT studies only (WMD = −0.42, 95% CI, −0.66, −0.17; *P* < 0.01) with moderate heterogeneity (*I*^2^ = 36%). Similar result was found in cohort studies (WMD = −0.35, 95% CI, −0.69, 0; *P*=0.05) (Figure 5). Meta-analysis revealed that postoperative side-to-side difference in anterior laxity was smaller in the remnant-preserving ACLR group than in the standard group.

**Figure 6:**
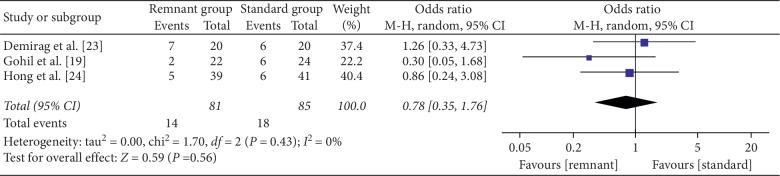
Forest plot for Lachman test. CI: confidence interval; MH: Mantel–Haenszel.

Lachman test was reported in four studies [19, 23, 24]. No difference was found in Lachman test between groups (OR = 0.78, 95% CI, 0.35, 1.76; *P*=0.56) (Figure 6).

**Figure 7:**
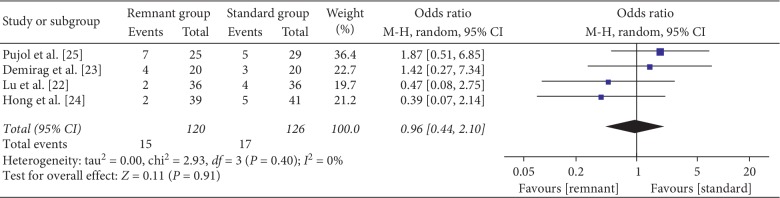
Forest plot for pivot-shift test. CI: confidence interval; MH: Mantel–Haenszel.

Pivot-shift test was reported in three studies [22–25]. No difference was found between groups respect to pivot-shift test (OR = 0.96, 95% CI, 0.44, 2.10; *P*=0.91) (Figure 7).

### 3.6. Status of Graft

Revascularization of the graft was evaluated by MRI in two studies. One study [15] reported similar maturation scores and tibial tunnel integration scores between groups. Another study [19] indicated a significant reduction in the midsubstance signal in the R group at 2 and 6 months postoperatively. Two studies [21, 23] reported better tibial tunnel widening in radiographs in the R group than in the C group.

**Figure 8:**
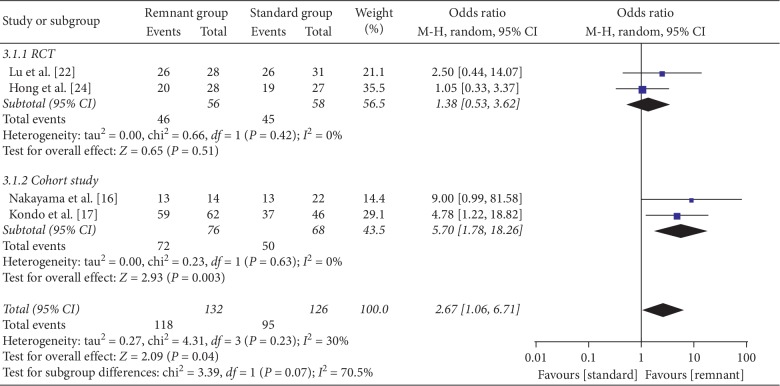
Forest plot for second-look arthroscopic evaluation. CI: confidence interval; MH: Mantel–Haenszel.

The graft status was evaluated by second-look arthroscopy in four studies (2 RCT and 2 cohort studies) [16, 17, 22, 24]. Several different methods were reported in previous studies to evaluate graft quality by second-look arthroscopy [33, 35, 36]. The main point of all those methods focuses on laceration of graft and synovial coverage and evaluates the score accordingly. Therefore, superior results were defined as grade A (grades A, B, and C) proposed by Kondo and Yasuda [35] or good (good, fair, and poor) proposed by Ochi et al. [33]. There was no significant difference in RCT studies between the two groups (OR = 1.38, 95% CI, 0.53, 3.62; *P*=0.51). A significant difference was found in cohort studies (OR = 5.7, 95% CI, 1.78, 18.26; *P*=0.003) with low heterogeneity (*I*^2^ = 0%), in favor of the R group (Figure 8).

### 3.7. Complications

**Figure 9:**
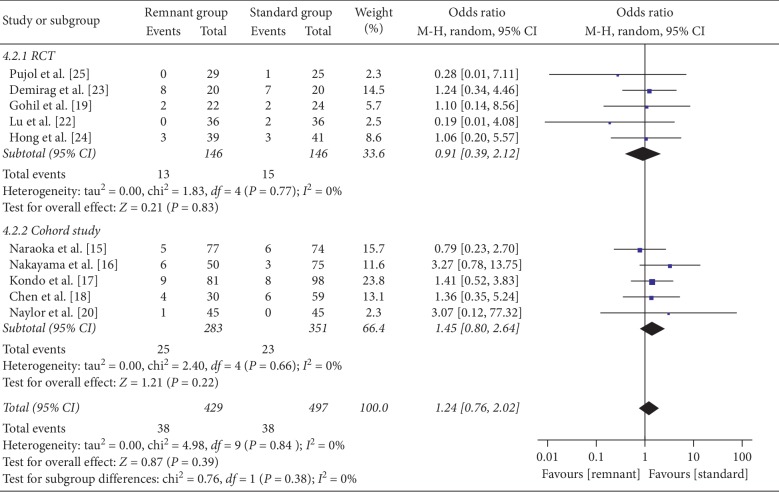
Forest plot for complication incidence. CI: confidence interval; MH: Mantel–Haenszel.

The overall complication rate was 8.2% (*n* = 38) in the remnant-preserving ACLR group (*n* = 466) and 7.1% (*n* = 38) in the standard ACLR group (*n* = 536). Complications that have been reported include range-of-motion (ROM) deficit, cyclops lesion, and knee instability. Of these complications, 84% (*n* = 64) were related to a ROM deficit (50%, *n* = 32) or cyclops lesion (50%, *n* = 32). There was no significant difference in RCT studies between the two groups with respect to complications (OR = 0.91, 95% CI, 0.39, 2.12; *P*=0.83). And no significant difference was observed when combined all studies (OR = 1.24 95% CI, 0.76, 2.02; *P*=0.39) (Figure 9).

## 4. Discussion

The principal findings of this systematic review were as follows: (1) an superior outcome of postoperative knee anterior stability and Lysholm score in patients undergoing remnant-preserving ACLR compared with those undergoing standard ACLR; (2) a similar healing status of grafts during second-look arthroscopy in the remnant-preserving ACLR group than in the standard ACLR group; and (3) no significant difference in the overall complication rate between groups. The available evidence at present does not support the notion that remnant-preserving ACLR is significantly superior to standard ACLR.

Four weeks after ACLR, the synovium with blood vessels from the subpatellar fat pad and synovial tissue begins to cover the graft, which leads to the revascularization and survival of the graft [37]. ACL remnants retain a well-vascularized synovial sheet, numerous fibroblasts and myofibroblasts, and mechanoreceptors [7–9]. Animal studies have found that ACL remnants can accelerate the process of synovial coverage and revascularization and enhance the biomechanical properties of grafts [8, 14]. However, a significant proportion of clinical studies have reported that there is no difference in graft healing between remnant-preserving ACLR and standard ACLR [16]. Second-look arthroscopy is a good tool to evaluate graft healing by observing synovial coverage, graft tension, and the presence of partial tears and impingement, according to Kondo and Yasuda [35] and Lee et al. [36]. Second-look arthroscopy was performed in 4 studies included in this review [16, 17, 22, 24], and meta-analysis reported similar graft status in the R group than in the C group. Kondo et al. [17] reported that arthroscopic evaluations in remnant-preserving ACLR were significantly better than those in standard ACLR, which significantly affected postoperative knee stability. Lu et al. [22] reported a better arthroscopic evaluation score, faster ROM recovery, and higher subjective outcome scores in the remnant-preserving ACLR group. Nakayama et al. [16] found better arthroscopic evaluations but an increased incidence of postoperative extension loss in the remnant-preserving ACLR group. Hong et al. [24] reported no differences in arthroscopic evaluations and clinical outcomes between groups. Among the three studies [16, 17, 22] that reported a significantly better graft status in the R group, only Lu et al. [22] found better subjective knee function scores in the R group. The benefits of remnant-preserving ACLR may be potential and long-term accumulation while improving knee stability and reducing postoperative meniscus damage and osteoarthritic changes [17]. Perhaps with longer follow-up, some differences emerge which could be found in rerupture rates, subjective results, and posttraumatic arthritis. Therefore, more randomized controlled and long-term follow-up studies are needed to confirm these hypotheses.

This meta-analysis showed a superior outcome of postoperative knee anterior stability and Lysholm score in the remnant-preserving ACLR group compared with the standard ACLR group. However, there were no significant differences between the two groups in IKDC grade, IKDC scores, Lachman test, and pivot-shift test. We believe the biomechanical stability of the knee joint should be the primary purpose of performing a successful ACLR. Kondo et al. [17] reported that the remnant-preserving technique could significantly improve postoperative knee stability by increasing the initial graft coverage. Kondo et al. believe that remnant preservation may enhance the biomechanical properties of the graft, which may affect the long-term clinical results concerning postoperative meniscus damage and/or osteoarthritic changes. Lu et al. [22] also reported a better arthroscopic evaluation score and knee anterior stability in the remnant-preserving ACLR group. However, Hong et al. [24] suggested the dominant postoperative stability was provided by the ACL graft itself, and the strength of the remnant may not be large enough to contribute a significant difference. In addition, the postoperative tension of the remnant was not adequately maintained, as shown in cases of abnormal synovial coverage. So conducting both second-look arthroscopy and KT arthrometer in RCT might help us to clarify the correlation. Many studies have reported that mechanoreceptors in the ACL remnant can promote reinnervation and restoration of proprioception [10–13, 18]. However, few human studies [12, 24, 38, 39] have evaluated the effect of remnant preservation on the recovery of proprioception function, and these studies have shown inconsistent results. Only one study [24] included in this review evaluated proprioception with the passive angle reproduction (RPP) test designed by Barrett [40], and no significant difference was found between groups. Adachi et al. [38] previously reported a better proprioception function with the RPP test in the remnant-preserving ACLR group. Although RPP was used to evaluate proprioception after ACL reconstruction, its sensitivity and specificity still need to be improved. In addition, the knee proprioception system is complex and consists of mechanoreceptors located in the ligament, joint capsule, tendons, and muscles [41, 42]. Distinguishing the effect of remnant preservation on the restoration of proprioception is difficult. Therefore, more sensitive and specific equipment or systems need to be developed to assess the proprioceptive function of the knee.

Surgical timing is one of the key factors for graft healing. Several studies [9, 13, 43, 44] have reported a decreased number of mechanoreceptors in an ACL stump with the time from injury to surgery. In addition, several histological studies [45–48] have reported that the gene expression patterns of the ACL stump change from healing to fibering over time. Inokuchi et al. [49] suggest that ACL remnant preservation can promote and enhance tendon-bone healing in the early phase after injury. Ahn et al. [6] reported better graft synovial coverage and incorporation outcomes in the R group, with a shorter duration between injury and surgery. However, a meta-analysis [50] has suggested that the interval between injury and surgery does not affect clinical outcomes. At present, the optimal timing for remnant-preserving ACLR in the clinical setting is still not clear. The optimal graft choice of remnant-preserving ACLR remains controversial. The autograft has been the mainstay in standard ACLR for a lower donor site failure rate and good clinical outcomes [51–54]. However, few articles have compared the clinical outcomes of different graft types in remnant-preserving ACLR. In this review, most included articles [15–18, 20–23] reported good clinical outcomes using autografts in the remnant-preserving ACLR group. Hong et al. [24] chose allografts and reported similar outcomes of stability, functional scores, revascularization, and proprioceptive between groups. Notably, synthetic grafts without self-tissue sacrifice are also a good choice. Chen et al. [18] reported that clinical scores were statistically significantly higher at 6 months postoperatively with synthetics in the remnant preservation group than in the autograft group. At a mean of 10 years postoperatively, synthetics and hamstring autografts demonstrated similarly satisfactory outcomes. The relationship between the graft type and clinical outcomes after remnant-preserving ACLR should be further studied.

The remnant amount is another important factor. Lee et al. [12] reported that increasing the remnant amount can promote the restoration of proprioceptive function. Muneta et al. [55] compared the clinical outcomes of three groups (classified according to the remnant volume: ≤30, 35–55, and ≥60%) and found that the remnant volume was weakly correlated with the postoperative outcome. On the other hand, Nakayama et al. [16] indicated that a large remnant may increase the incidence of cyclops lesions and extension loss. The studies included in this systematic review rarely involve the remnant amount. Tibial tunnel widening is a common problem after ACL reconstruction [56–58]. Tibial tunnel widening could induce poor healing of graft because of infiltration of synovial fluid into the space between bone and graft. Previous studies [59, 60] have reported that remnants of ACL can cover the entry of the tibial tunnel and decrease the infiltration of synovial fluid. Three studies [15, 21, 23] included in this review measured tibial tunnel widening. Zhang et al. [21] and Demirag et al. [23] found few outcomes of tibial tunnel widening in the R group. The 2 studies measured tibial tunnel widening by radiography, which is not as accurate as computed tomography or MRI. In addition, these studies failed to find a correlation between tibial tunnel widening and knee joint stability at the final follow-up.

The main complications of the remnant-preserving technique were cyclops lesion and extension loss. This meta-analysis showed that there was no significant difference in the overall complication rate between groups. Nakayama et al. [16] reported increased extension deficits for knees with double-bundle remnant-preserving ACLR. The semitendinosus tendon that is folded in four for double-bundle ACLR needs a large volume, and the full volume of the preserved remnant with the suturing/tensioning technique described by Ahn et al. [32] takes up additional space. However, Kondo et al. [17] observed rare cyclops lesions in remnant-preserving ACLR with double-bundle ACLR that was similar to standard ACLR. Different remnant preservation techniques are also an influencing factor of the incidence of cyclops lesions. Selective-bundle augmentation is a different technique than standard ACLR with remnant preservation, which reconstructs a single-bundle (anteromedial bundle or posterolateral bundle) with the other bundle remnant preserved. Selective-bundle augmentation may have a smaller incidence of cyclops lesions because there are no excess remnant fibers. Kondo et al. [17] used the sparing technique in remnant-preserving ACLR, which reduces the volume of the remnant during the drilling and passage of the hamstring graft. This suggests that the sparing technique may have potential advantages over the tensioning technique in reducing the rate of cyclops lesions and extension deficits. However, partial or complete resection of preserved remnants can be considered for knees with narrow intercondylar fossa and large remnants [16].

## 5. Limitations

This review has several limitations. First, to include high-quality studies, the number of included studies and patients was relatively small. Second, some prospective cohort studies included in this review and meta-analysis have selection bias, including heterogeneity in patient populations, surgical techniques, and measures of clinical outcomes, which leads to higher heterogeneity when simultaneously combining randomized controlled trials and cohort studies in subgroup meta-analysis. Third, the median follow-up duration in the studies was approximately two years, and longer follow-up is needed to evaluate the difference between the two techniques. Fourth, single-bundle augmentation is a different technique than standard ACLR with remnant preservation. However, subgroup analysis of surgical technique comparison was not performed in this review because of the small number of studies and the high heterogeneity. More research is needed in the future to compare these technologies.

## 6. Conclusion

This systematic review showed that remnant-preserving ACLR promoted similar synovial coverage and revascularization of grafts to standard ACLR. Equivalent or superior outcomes of postoperative knee stability and clinical scores were observed in patients undergoing remnant-preserving ACLR compared with those undergoing standard ACLR. There was no significant difference in the rate of total complications between groups. Three different remnant-preserving techniques included in this review have respective advantages, and more research is needed in the future to compare these technologies. The currently available evidence is not sufficiently strong to support the superiority of remnant-preserving ACLR.

## Figures and Tables

**Table 1:** Distributions of factors related to final results within studies.

Study	Time from injury to surgery (mo)	Pattern of ACL rupture	Amount of remnant	Type of graft	Procedure
Pujol et al [25]	Group R: 4.5 Group C: 5	Group R: partial Group C: partial	Length: bridging femur and tibia in group R No remnant left in group C	Group R: auto Group C: auto	Group R: selective-bundle augmentation Group C: standard ACLR

Zhang et al. [21]	Group R: 12.7 Group C: 10.4	Group R: complete Group C: complete	Diameter: intact tibial remnant observed in both groups	Group R: auto Group C: auto	Group R: standard ACLR + tibial remnant sparing Group C: standard ACLR

Demirag et al. [23]	Group R: 2.3 Group C: 8.0	Group R: partial Group C: partial	Length: bridging femur and tibia in both groups Diameter: >50% of native ACL in both groups	Group R: auto Group C: auto	Group R: selective-bundle augmentation Group C: standard ACLR

Gohil et al. [19]	Group R: 1.9 Group C: 2.4	NA	NA	Group R: auto Group C: auto	Group R: standard ACLR + tibial remnant sparing Group C: standard ACLR

Lu et al. [22]	Group R: 0.8 Group C: 0.8	Group R: complete Group C: complete	Length: bridging femur and tibia in group R No remnant left in group C	Group R: auto Group C: auto	Group R: standard ACLR + tibial remnant tensioning Group C: standard ACLR

Hong et al. [24]	Group R: 10.3 Group C: 9.4	Group R: complete Group C: complete	Length: able to be pulled into femoral tunnel and in both groups Diameter: >50% of native ACL in both groups	Group R: allo Group C: allo	Group R: standard ACLR + tibial remnant tensioning Group C: standard ACLR

Naraoka et al. [15]	NA	NA	Length: >25% of native ACL in group R <25% of native ACL in group C	Group R: auto Group C: auto	Group R: standard ACLR + tibial remnant tensioning Group C: standard ACLR

Nakayama et al. [16]	Group R: 12 Group C: 12	Group R: complete Group C: complete	Diameter: ≥50% of native ACL in group R No remnant left in group C	Group R: auto Group C: auto	Group R: standard ACLR + tibial remnant tensioning Group C: standard ACLR

Kondo et al. [17]	Group R: 7 Group C: 12	Group R: complete Group C: complete	Length: bridging femur and tibia in group R No remnant left in group C	Group R: auto Group C: auto	Group R: standard ACLR + tibial remnant tensioning Group C: standard ACLR

Chen et al. [18]	Group R: 16.5 Group C: 18	Group R: complete Group C: complete	NA	Group R: LARS Group C: auto	Group R: standard ACLR + tibial remnant sparing Group C: standard ACLR

Rushton et al. [20]	<3 mo: R 21, C 16 <3 mo: R 24, C 29	Group R: complete + partial Group C: complete + partial	NA	Group R: auto Group C: auto	Group R: standard ACLR + tibial remnant sparing Group C: standard ACLR

Allo, allograft; auto, autograft; R, remnant; C, control; NA, not available; mo, month; LARS, Ligament Augmentation Reinforcement System.

**Table 2:** Distributions of factors related to final results within studies.

Study	Number of patients	Follow-up (mo)	Outcome measure	Results	Complications	Conclusion
Pujol et al [25].	Group R: 29 Group C: 25	Group R: 12 Group C: 12	IKDC, Lysholm, KOOS, Rolimeter® knee tester	IKDC, Lysholm, and KOOS improved in both groups Anterior laxity on Rolimeter®: 1.24 mm in group R vs. 1.87 mm in group C	Cyclops lesions: 1 in group R	No difference in functional scores between groups Better short-term control of anterior laxity in group R than in group C

Zhang et al. [21]	Group R: 27 Group C: 24	Group R: 24.4 Group C: 25.2	Lysholm, KT-1000 knee arthrometer Radiographs: tibial tunnel widening	Lysholm and KT-1000 improved in both groups Tibial tunnel widening: 12.9 ± 1.0 mm in group R vs. 13.9 ± 1.3 mm in group C	NA	No difference in functional score and joint stability between groups Better tibial tunnel widening outcome in group R

Demirag et al. [23]	Group R: 20 Group C: 20	Group R: 24.3 Group C: 24.3	IKDC and Lysholm Radiographs: tibial tunnel widening	IKDC and Lysholm improved in both groups Tibial tunnel widening: 7.7 ± 0.5 mm in group R vs. 7.9 ± 0.5 mm in group C	Flexion loss: 7 in each group Cyclops lesions: 1 in group R	No differences in functional scores between groups Better tibial tunnel widening outcome in group R

Gohil et al. [19]	Group R: 24 Group C: 25	Group R: 12 Group C: 12	IKDC, KT-1000 knee arthrometer One-legged hop test MRI	IKDC and KT-1000 improved in both groups MRI: significant reduction in midsubstance signal in group R at 2 and 6 mo postoperatively	Extension loss: 2 in each group	Earlier revascularization in group R than in group C

Lu et al. [22]	Group R: 36 Group C: 36	Group R: 34.7 Group C: 39.6	IKDC, Lysholm, Tegner, KT-2000 knee arthrometer Second-look arthroscopy	Better outcomes of IKDC, Lysholm, and KT-2000 in group R than in group C Evaluation of graft quality on arthroscopy: 4.6 ± 1.6 scores in group R vs. 3.9 ± 2.0 scores in group C	Knee instability: 2 in group C	Faster ROM recovery, higher subjective outcome scores, and better second-look arthroscopy in group R than in group C

Hong et al. [24]	Group R: 39 Group C: 41	Group R: 25.8 Group C: 25.5	IKDC, Lysholm, KT-1000 knee arthrometer Second-look arthroscopy Proprioceptive testing: RPP test	IKDC, Lysholm, and KT-1000 improved in both groups Second-look arthroscopy: >50% graft synovial coverage in 20/28 in group R vs. 19/27 in group C RPP test: 3.6° ± 1.8° in group R vs. 3.9° ± 2.2° in group C	Cyclops lesions: 3 in each group	No differences in stability, functional scores, revascularization, and proprioceptive outcomes between groups

Naraoka et al. [15]	Group R: 77 Group C: 74	Group R: 24 Group C: 24	Lysholm, KT-1000 knee arthrometer Magnetic resonance imaging: MRI	Lysholm and KT-1000 improved in both groups MRI: similar result of maturation scores and tibial tunnel integration scores between groups	Rerupture: 5 in group R and 6 in group C	No difference in stability and graft incorporation between groups

Nakayama et al. [16]	Group R: 50 Group C: 75	Group R: 12 Group C: 12	Heel height difference, Lysholm, KT-1000 knee arthrometer Second-look arthroscopy	Lysholm and KT-1000 improved in both groups Second-look arthroscopy: 92% good status of grafts in group R vs. 59% good in group C	Extension loss: 6 in group R and 3 in group C	Better tissue healing but higher incidence of extension loss in group R than in group C

Kondo et al. [17]	Group R: 81 Group C: 98	Group R: 24 Group C: 24	IKDC, Lysholm, KT-2000 knee arthrometer 3-Dimensional computed tomography: 3D-CT Second-look arthroscopy	No difference in IKDC and 3D-CT between groups Anterior laxity on KT-2000 : 43/81 < 1 mm in group R vs. 33/98 < 1 mm in group C Second-look arthroscopy: excellent status of grafts in 59/81 in group R vs. 37/98 in group C	Cyclops lesions: 9 in group R and 8 in group C (no symptoms)	Postoperative knee stability significantly improved in group R

Chen et al. [18]	Group R: 38 Group C: 73	Group R: 120.8 Group C: 122.9	IKDC, Lysholm, KOOS, Tegner Radiography	Better scores of IKDC, Lysholm, KOOS, and Tegner in group R than in group C at 6 mo postoperatively	Screw-related problem: 3 in R and 2 in C Donor site morbidity: 3 in C Superficial infection: 1 in C Synovitis: 1 in R	Earlier symptom relief and restoration of function in group R than in group C

Rushton et al. [20]	Group R: 45 Group C: 45	Group R: 12 Group C: 12	IKDC, KT-2000 knee arthrometer ACL-QOL (ACL-quality of life)	IKDC improved in both groups ACL-QOL: improved scores of 54.7 in group R vs. 46.1 in group C Side-to-side difference on KT-2000 : 2.1 ± 1.3 mm in group R vs. 3.0 ± 1.7 mm in group C	Cyclops lesions:1 in group R	Better knee stability and quality of life in group R than in group C

R, remnant; C, control; NA, not available; IKDC, International Knee Documentation Committee; KOOS, Knee Injury and Osteoarthritis Outcome Score.

**Table 3:** Study features.

Study	Publication year	Country	Type of study	Level of evidence	CMS score
Pujol et al. [25]	2012	France	Randomized controlled trial	I	79
Zhang et al. [21]	2012	China	Randomized controlled trial	I	89
Demirag et al. [23]	2012	Turkey	Randomized controlled trial	I	87
Gohil et al. [19]	2007	Australia	Randomized controlled trial	I	91
Lu et al. [22]	2015	China	Randomized controlled trial	II	92
Hong et al. [24]	2012	China	Randomized controlled trial	II	92
Naraoka et al. [15]	2017	Japan	Prospective cohort study	II	87
Nakayama et al. [16]	2017	Japan	Prospective cohort study	II	84
Kondo et al. [17]	2015	Japan	Prospective cohort study	II	77
Chen et al. [18]	2012	China	Prospective cohort study	II	87
Rushton et al. [20]	2012	Canada	Prospective cohort study	II	79

CMS score, the modified Coleman methodology score.

**Table 4:** Detailed CMS for included studies.

Study	Publication year	Study size	Mean follow-up	Number of procedures	Type of study	Diagnostic certainty	Surgery description	Rehabilitation description	Outcome criteria	Procedure for outcome	Selection process	Total score
Pujol et al. [25].	2012	7	2	10	15	5	3	0	10	12	15	79
Zhang et al. [21]	2012	7	5	10	15	5	5	10	10	12	10	89
Demirag et al. [23]	2012	4	5	10	15	5	5	0	10	12	15	87
Gohil et al. [19]	2007	7	2	10	15	5	5	10	10	12	15	91
Lu et al. [22]	2015	10	5	10	10	5	5	10	10	12	15	92
Hong et al. [24]	2012	10	5	10	15	5	5	10	10	12	10	92
Naraoka et al. [15]	2017	10	5	10	10	5	5	10	10	12	10	87
Nakayama et al. [16]	2017	10	2	10	10	5	5	10	10	12	10	84
Kondo et al. [17]	2015	10	5	10	10	5	5	0	10	12	10	77
Chen et al. [18]	2012	10	5	10	10	5	5	10	10	12	10	87
Rushton et al. [20]	2012	10	2	10	10	5	5	0	10	12	15	79

**Table 5:** Descriptions of 3 different remnant preservation techniques.

Tibial remnant tensioning [32]	Selective-bundle augmentation [33]	Tibial remnant sparing [34]
Several sutures of the remnant ACL were placed near the proximal end.	When the ACL remnant was attached to the anteroinferior portion of the anatomic femoral origin and the posterolateral (PL) bundle was well preserved, the anteromedial (AM) bundle was reconstructed.	The tibial tunnel position was within the boundaries of the ACL tibial remnant.

Medial traction of these sutures provided a wide view during the reconstruction.	When the ACL remnant was attached to the high-noon position with a well-preserved AM bundle, the PL bundle was reconstructed.	The ACL graft was allowed to pass through the tibial tunnel within the tibial remnant.

Fixation was performed with a slightly smaller tension with the tibial remnant from the femoral tunnel.		
